# A cost−utility study of elective haemorrhoidectomies in Canada

**DOI:** 10.1111/codi.16867

**Published:** 2024-01-21

**Authors:** M. Guo, A. A. Karimuddin, G. Liu, T. Crump, C. J. Brown, M. J. Raval, P. T. Phang, A. Ghuman, J. Mok, J. M. Sutherland

**Affiliations:** ^1^ Department of Surgery University of British Columbia Vancouver British Columbia Canada; ^2^ Department of Colorectal Surgery St Paul's Hospital Vancouver British Columbia Canada; ^3^ Centre for Health Services and Policy Research University of British Columbia Vancouver British Columbia Canada; ^4^ Department of Surgery McGill University Montreal Quebec Canada; ^5^ Department of Family Medicine Queen's University Kingston Ontario Canada

**Keywords:** cost‐utility, haemorrhoidectomy, health, quality‐adjusted life years

## Abstract

**Aim:**

The aim was to estimate the 10‐year cost−utility of haemorrhoidectomy surgery with preference‐based measures of health using Canadian health utility measures and costs.

**Methods:**

Patients undergoing elective haemorrhoidectomies by general and colorectal surgeons in British Columbia, Vancouver, between September 2015 and November 2022, completed preoperatively and postoperatively the EuroQol five‐dimension five‐level health‐related quality of life questionnaire (EQ‐5D‐5L). Quality‐adjusted life years (QALYs) attributable to surgery were calculated by discounting preoperative and postoperative health utility values derived from the EQ‐5D‐5L. Costs were measured from a health system perspective which incorporated costs of hospital stay and specialists' fees. Results are presented in 2021 Canadian dollars.

**Results:**

Of 94 (47%) patients who completed both the preoperative and postoperative questionnaires, the mean gain in QALYs 10 years after surgery was 1.0609, assuming a 3.5% annual discounting rate. The average cost of the surgery was $3166. The average cost per QALY was $2985 when benefits of the surgery were assumed to accrue for 10 years. The cost per QALY was higher for women ($3821) compared with men ($2485). Participants over the age of 70 had the highest cost per QALY ($8079/QALY).

**Conclusions:**

Haemorrhoidectomies have been associated with significant gains in health status and are inexpensive relative to the associated gains in quality of life based on patients' perspectives of their improvement in health and well‐being.

**What does this paper add to the literature?**

Valuing the benefits of haemorrhoidectomies on patients' postoperative well‐being relative to the surgery's cost is important to the allocation of healthcare resources. This study provides a perspective of the intervention's cost relative to the value of the surgery from a patient perspective, a novel use of economic principles applied to surgery.

## INTRODUCTION

Haemorrhoids, or the engorgement of vascular structures near the anal canal, are one of the most common medical conditions affecting the general population [1]. Whilst precise estimates of the prevalence of haemorrhoids are uncertain, a recent internationally administered online survey estimated the prevalence of haemorrhoidal disease to be 11% [2], although this may be underestimated due to under‐reporting.

Haemorrhoids are often associated with a large negative impact on patients' quality of life, with an estimated annual cost of $770 million in the United States (in 2014 dollars) for its treatment and complications [3]. Whilst many patients with haemorrhoids can be treated with non‐operative methods, failure of non‐operative methods or the manifestation of complications prompt surgical intervention [4]. Hence, the overall costs of operative treatment of haemorrhoids are substantial due to the high prevalence of disease.

Gains in quality of life and well‐being attributable to haemorrhoid surgery have been demonstrated amongst patients undergoing a range of surgical techniques for haemorrhoid removal [5, 6, 7]. Long‐term follow‐up using patient‐reported outcome measures has shown sustained improvements in disease‐related symptoms for over 10 years [8, 9]. However, the gains in quality of life vis‐à‐vis the surgery's costs are poorly understood [7, 10, 11]. There is some literature comparing the cost‐effectiveness between different surgical techniques [6, 7, 12], with reporting of improvement in quality of life measures on short‐term follow‐up. However, there are no studies weighing the long‐term benefits with costs attributable to the surgical treatment of haemorrhoids. In addition, there is a need for country‐specific cost‐utility analyses since costs of equipment and labour, as well as criteria for operative management, differ by setting. These factors may contribute to differences in observed quality‐adjusted life years (QALYs) among patients [13, 14], which are critical to translating the economic benefits of the surgery [13].

The impact of haemorrhoid surgery can be expressed in terms of QALYs, which are estimations of both quality and duration of life after an intervention. The quality component of QALYs can be inferred from gains in health utility measured with multi‐attribute utility instruments, such as the EuroQol five‐dimension five‐level health‐related quality of life questionnaire (EQ‐5D‐5L). Calculations of quality improvements when projected over time are typically discounted to reflect time‐based decline in health preference.

The purpose of this study is to estimate the cost−utility of haemorrhoidectomy using a preference‐based multi‐attribute utility instrument that measures health status preoperatively and postoperatively. Empirical data generated by this study provide surgeons and health insurers with insights into the gains in their patients' health attributable to their surgery over different periods of time. The information generated from this study would be valuable to policy‐makers in assessing existing interventions and allocating incremental increases in surgical capacity.

## METHODS

### Study design

This study was based on retrospective analysis of a longitudinal cohort of consecutive patients prospectively recruited prior to their haemorrhoid surgery in Vancouver Coastal Health Authority, an urban region that includes Vancouver, Canada. All consecutive haemorrhoid surgery patients of six colorectal surgeons in one acute teaching hospital who were scheduled for their surgery were eligible to participate. Patients had to be 19 years of age or older, living in the community and not a conjugate setting such as a nursing home, scheduled for surgery at least 2 weeks from being enrolled on the surgical registry to remove emergent cases, and able to respond to the survey with or without assistance in English.

Eligible patients were contacted by phone to participate prior to their surgery. Patients who agreed to participate were sent a survey package through the mail or an email with a link to complete a web‐based version. The survey package included a covering letter describing the survey and instructions to complete the survey EQ‐5D‐5L, a preference‐based multi‐attribute utility instrument [15]. Participants were contacted after their surgery by mail or email and completed the EQ‐5D‐5L 6 months postoperatively.

Participants were deterministically linked with population‐based hospital discharge summary data and emergency department visits occurring up to 6 months after discharge from their haemorrhoid surgery. Hospital discharge summary data provided information regarding sex, age at surgery and comorbidities. The study was based on participants completing postoperative surveys between September 2015 and November 2022. The University of British Columbia's Behavioural Research Ethics Board approved the study (H21‐02641).

### Outcome measures

The EQ‐5D‐5L is a survey that asks patients to rate five domains related to their quality of life, including mobility, self‐care, usual activities, pain/discomfort and anxiety/depression. Patients rate each domain on a five‐point scale, which translates to 3125 unique health states. The pattern of responses generates a participant's health state [16]. The health state corresponds to a unique health utility value determined independently of this study [17, 18]. The instrument also includes a visual analogue scale not used in this study.

The utility values represent a preference‐based measure of health as measured by the EQ‐5D‐5L. The values range from −0.34 to 1. Values below 0 represent a health state considered worse than death, while a utility value of 1 represents perfect health [17]. Considered over time, utility values provide a means to calculate QALYs [19] as the product of utility values and duration of observation. For example, 1 year at perfect health and a utility value of 1 translates to one QALY.

In this study, each participant's health state was linked with the EQ‐5D‐5L's utility values for each of the preoperative and postoperative survey points [20, 21]. The EQ‐5D‐5L questionnaire was mailed or emailed to agreeing participants before their surgery date. Participants completed the same instrument 6 months after their surgery. Health state utility values of participants were summarized with mean, standard deviation and range statistics at the preoperative and postoperative time points. The change between preoperative and postoperative utility values was evaluated through a paired *t* test.

### Calculating quality‐adjusted life years (QALYs)

This analysis calculated QALYs using an approach adapted from elsewhere, including the UK's National Health Service [22, 23] and in Canada [24, 25], to measure gains attributable to surgery. Preoperative utility values estimated the patient's quality of life before haemorrhoidectomy, while postoperative utility values reflected their quality of life after the procedure. A 10‐year duration of health improvement from the procedure was assumed based on the longest reported follow‐up time on symptom control following haemorrhoidectomy [8, 26].

Discounting was applied to projected utility scores over the 10‐year interval to account for the effect of current time preference on health status [23]. With these assumptions, QALYs were calculated as the difference between the preoperative and postoperative discounted health utility values over the 10‐year time period. The sensitivity of the findings due to the utility values' discount rate was evaluated at both 1.5% and 3.5% per year, consistent with the Canadian Agency for Drugs and Technologies in Health and the UK's National Institute for Clinical Excellence recommendations, respectively [26, 27].

### Calculating cost per quality‐adjusted life year (QALY)

This study assumed a health systems perspective of measuring cost per QALY. The cost data were based on identifying direct public spending on the surgery and complications, consistent with provision of hospital and physician services being free at the point of access, in this setting. Costs attributable to the surgery did not include patient‐borne or indirect costs, such as lost productivity.

Each participant's cost was calculated as the sum of hospital‐borne costs of the surgery, the surgeon's fee‐for‐service charges for the operation, and associated anaesthesia costs assuming an uncomplicated case [28]. Hospital costs were derived from participants' hospitalization's case mix adjusted cost weight, provided independent of this study by the Canadian Institute for Health Information, and the hospital's cost per weighted case [29]. All costs were measured in 2021 Canadian dollars.

Each participant's cost also included potential complications related to the operation, including emergency department visits or rehospitalizations within 6 months of discharge and reflecting clinically relevant healthcare commensurate with the surgery and complications requiring intervention.

Cost per QALY statistics were calculated at the participant level as participants' cost divided by their QALYs. Uncertainty regarding participant's cost per QALY was quantified through application of non‐parametric bootstrap methods, resampling participants' data 200 times [30]. To explore age‐ and sex‐based differences in cost per QALY statistics, the same process was applied for calculating confidence intervals among subgroups. This exploratory analysis was not designed to test for statistical differences among participants' characteristics. As a result, the sample size was not determined a priori. All analyses were conducted using SAS, version 9.4 [31].

## RESULTS

Among 293 eligible patients, 138 participants (47%) completed the preoperative survey. Among these participants, 68% also completed the postoperative survey, providing this study with a sample of 94 participants. Participants were on average 3 years older than non‐participants. No sex‐based difference in participation rate was observed.

As shown in Table 1, 55.3% of participants were women and 57.4% had at least one comorbidity. There was a statistically significant gain in utility values to the postoperative mean of 0.8935 (*P* < 0.01). All participants underwent traditional haemorrhoidectomy (either open or closed), as stapler‐based excision was not standard of care at the study's institution. Figure 1 illustrates the distribution of the sample's preoperative and postoperative health utility values.

**TABLE 1:** Summary characteristics of participants, based on profile prior to their surgery and answers to patient‐reported outcomes at 6 months after surgery.

Characteristics	*N* (%)
Sex	
Male	42 (44.7)
Female	52 (55.3)
Age group	
19–40	13 (13.8)
41–55	24 (25.5)
56–70	46 (48.9)
71–81	11 (11.7)
Comorbidities	
0	40 (42.6)
1–2	42 (44.7)
>2	12 (12.7)
Utility value	
Preoperative (mean ± SD, range)	0.7967 ± 0.1764 (0.1011, 0.9926)
Postoperative (mean ± SD, range)	0.8935 ± 0.1590 (0.0451, 0.9926)
*P* value	<0.0001

**FIGURE 1:**
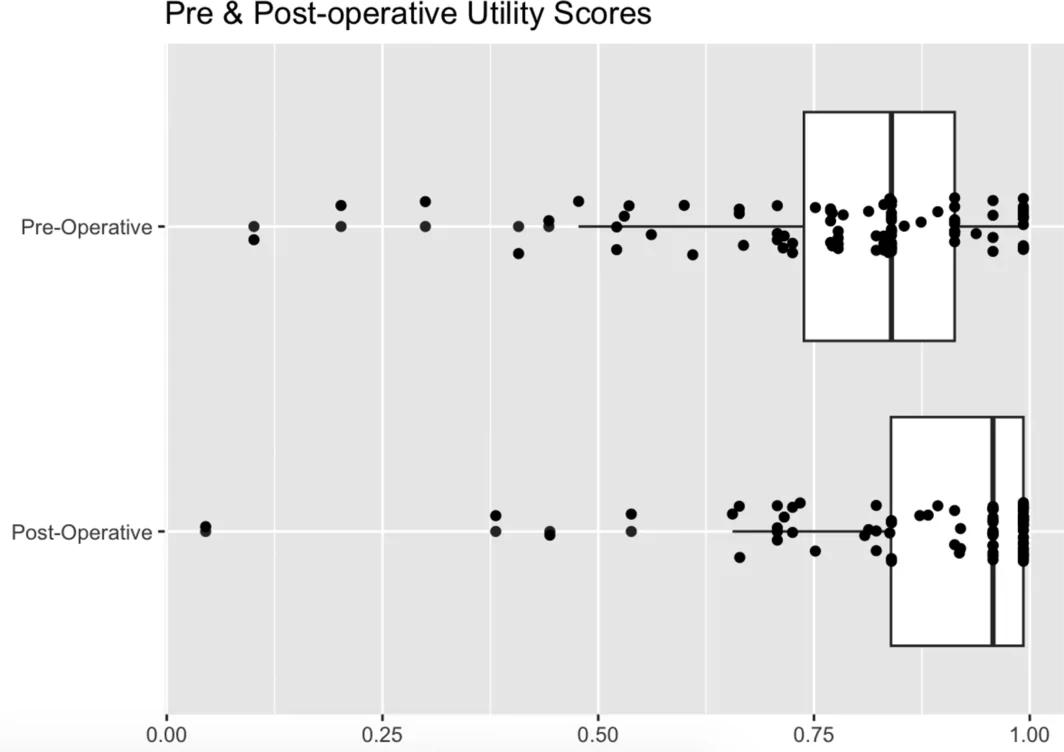
Comparison of participants' preoperative and postoperative EQ‐5D‐5L health utility values. The superimposed boxplot illustrates the mean and interquartile range of utility values.

A higher number of comorbidities was observed in the oldest age group (71–81 years) at 2.6 comorbidities per person, compared with 1.1, 0.7 and 1.1 in the 56–70 years, 41–55 years and 19–40 years age categories, respectively. One relevant emergency department visit and rehospitalization was identified in the entire cohort within 6 months of surgery, which was for postoperative bleeding.

Accounting for health gains accrued for 10 years after surgery, the haemorrhoidectomy's average cost per QALY was $2985 when health utilities were discounted at an annual rate of 3.5%, and $2943 when the annual discount rate of 1.5% was applied (Table 2). The study found smaller gains in QALYs among women than men on average. An age‐based gradient was also observed in the mean cost per QALY. Participants over the age of 70 had a higher cost per QALY ($8079/QALY) than patients aged 19–70 years (Table 2). The only surgical complication identified within 6 months of surgery was for postoperative bleed of a participant in the oldest age category.

**TABLE 2:** Mean quality of life years (QALYs) attributed to participants, mean costs of surgery and mean cost per QALY among all participants assuming gains in health were realized for 10 years following haemorrhoidectomy.

	Mean gain in QALYs (SD)	Mean hospital and specialists’ cost	Mean cost per QALY	95% confidence interval^a^
*1.5% discounting*				
Overall	1.0761 (1.4300)	3166 (2575)	2943	(2173, 4307)
Sex				
Male	1.2206 (1.5989)	2983 (1186)	2444	(1787, 3767)
Female	0.8972 (1.1828)	3393 (3633)	3782	(2393, 6839)
Age category				
19–40	1.3960 (1.6762)	2779 (208)	1990	(1158, 4574)
41–55	1.4839 (1.9952)	3446 (1836)	2322	(1374, 4188)
56–70	0.8861 (0.9866)	2710 (138)	3058	(2336, 4548)
71–81	0.6030 (1.0795)	4924 (7004)	8165	(2409, 63563)
***3**.5% discounting*				
Overall	1.0609 (1.3142)	3166 (2575)	2985	(2241, 4254)
Sex				
Male	1.2006 (1.4663)	2983 (1186)	2485	(2475, 6737)
Female	0.8880 (1.0899)	3393 (3633)	3821	(1856, 3680)
Age category				
19–40	1.3539 (1.5292)	2779 (208)	2052	(1305, 4743)
41–55	1.4185 (1.8357)	3446 (1836)	1836	(1478, 4696)
56–70	0.8996 (0.9150)	2710 (138)	3012	(2278, 4225)
71–81	0.6094 (0.9880)	4924 (7004)	8079	(2736, 40333)

*Note*: Results are stratified by sex and age categories.

^a^ Generated through non‐parametric bootstrap methods, resampling participants’ data 200 times.

## DISCUSSION

This was the first cost−utility study on elective haemorrhoidectomies to quantify gains in quality of life using a 10‐year time horizon. The results of this study provide an empirical basis for understanding the quality of life benefits of a haemorrhoidectomy versus the costs associated with hospital utilization and clinician fees. For surgeons operating with constrained resources, the data can be used to objectively understand the ‘worth’ of a haemorrhoidectomy to an average patient, which can be incorporated into decision‐making when allocating elective operating time.

The participants of this study demonstrated significant gains in health utility 6 months after their haemorrhoidectomy. This finding was consistent with results generated from earlier prospective randomized trials in England and the United States, although the results were highly variable [6, 7, 12]. Authors of the eTHoS trial comparing traditional technique to staple technique haemorrhoidectomies reported a mean increase of 1.69 QALYs at 1 year after traditional haemorrhoidectomy [7]. Similarly, Kilonzo et al. reported a gain of 1.738 QALYs over 2 years after traditional haemorrhoidectomies [12] while Ribarić et al. reported a gain of 0.76 after 1 year of follow‐up after traditional haemorrhoidectomies [6]. Cost estimates for classic ‘open’ or ‘closed’ approaches are fairly consistent in the most recent studies [5, 6, 7, 32]. Our results provide a long‐term perspective of the intervention costs relative to the value of the surgery from a patient perspective, a novel use of health economic principles applied to surgical procedures.

The study found the average cost per QALY was $2985 CAD assuming benefits from haemorrhoidectomies accrued for 10 years; this is an amount that compares favourably with cost‐effectiveness thresholds used for evaluating new therapies at $50,000 CAD [32]. Subgroup analysis found that the cost per QALY was highest among the oldest participants. This observation is probably attributed to higher overall costs associated with the procedure and less QALYs gained from the surgery. The smaller gains in QALYs among older participants may have reflected the presence of concomitant comorbidities and functional limitations that made postoperative recovery more challenging and gain in health status more limited [33]. This hypothesis was supported by our data, which showed that there were more than double the number of comorbidities among participants in the oldest age category compared with younger participants. Taken together, these results may provide an evidentiary basis for further study regarding the benefit in health status of providing haemorrhoid surgery to those with severe comorbidities and unlikely to see improvement in quality of life or short life expectancy. However, viewpoints on the safety and efficacy of haemorrhoidectomies in the elderly and comorbid have been conflicting, with small studies showing comparative outcomes between the young and elderly [34].

Cost per QALY was higher for women than for men despite comparable costs. Sex‐based differences in QALY gains have not been reported in prior studies, owing to a lack of physiological or pathophysiological basis for sex‐based differences in disease or surgical outcomes. It is possible that this study drew a sample of men who had, on average, a higher threshold for healthcare seeking behaviours for haemorrhoidal disease and other anorectal diseases, which led to poorer health status at time of presentation [35, 36], resulting in larger improvements after surgery.

Based on our sample of 94 participants, the low cost per QALY for haemorrhoidectomies based on Canadian cost data suggests that haemorrhoidectomies were a sound investment of public healthcare funding. Results were comparable with other surgical procedures. For example, previously published cost−utility analyses of elective cholecystectomies using a similar methodology showed a similarly low cost per QALY of $2102 in 2021 Canadian dollars, albeit assuming health benefits accrued for 25 years [24]. In the setting of this study, provincial governments were stewards of public funds used to operate hospital care that is free to residents at point of access and these results may serve as one point of evidence supporting incremental investments in increasing surgical capacity.

### Assumptions and limitations

The study was conducted from the healthcare funder's viewpoint and did not consider patient or societal costs, such as lost income or privately paid medical expenses. Hence, we may be underestimating the benefit of the operation in relation to its costs when viewed from the societal perspective. Consequently, these findings may serve to establish a minimum benchmark for assessing the value of haemorrhoidectomies. Canadian costs and Canadian‐derived utility values are inputs to this study which may lessen the generalizability of the results to other countries. However, we anticipate variations to be small given the standardized decision‐making and surgical approach for hemorrhoidectomies plus demonstrated consistency in quality‐of‐life improvements across different countries [7, 12].

Due to the limited sample size and low adverse event rate, the full impact of adverse events on quality of life may not have been reflected in this study. This was due to a low adverse event rate and provides a basis for expanding the study's inclusion criteria to other recruitment sites. Also, the study focused on surgical haemorrhoidectomies, and results may not be extrapolated to office‐based procedures such as rubber band ligation.

Given the observational and voluntary nature of this study, participants may not have been representative of the population of haemorrhoidectomy patients. For example, non‐English speakers were excluded from the study. While this study could not identify relevant family physician visits, all emergency department visits and hospitalizations that occurred during the 6‐month post‐discharge period were included. Finally, this study assumed that health states after surgery were attributable to the surgery itself and not due to changes attributable to other interventions. This was considered a reasonable assumption, as the patient cohort was predominantly healthy with few comorbidities.

For clinicians involved in allocating surgical resources, the findings from this study may be useful for informing or supporting advocacy for increased access to haemorrhoidectomies. Additionally, smaller relative gains in improvements in health status among elderly patients may shift some discussion to focusing on alternative and complementary approaches to improving health and well‐being among elderly patients. Furthermore, the higher quality of life gains observed in men before surgery might suggest differences in symptom severity communication between genders, which could be addressed through new communication strategies in clinical settings.

## CONCLUSION

Participants undergoing haemorrhoidectomies reported significant gains in health‐related quality of life after the surgery. The costs of the intervention were found to be relatively inexpensive when accounting for the associated gains in quality of life based on participants' perspectives of their improvement in health status. This study provides an empirical basis for future cost‐effectiveness studies exploring the value of haemorrhoidectomies from a patient perspective, as well as policy‐driven queries on the value provided by different surgeries.

## AUTHOR CONTRIBUTIONS

**J. M. Sutherland:** Conceptualization; investigation; funding acquisition; writing – original draft; methodology; writing – review and editing; project administration; supervision; resources. **M. Guo:** Conceptualization; investigation; writing – original draft; writing – review and editing; validation. **A. A. Karimuddin:** Conceptualization; investigation; writing – original draft; writing – review and editing. **G. Liu:** Funding acquisition; writing – original draft; methodology; validation; formal analysis; supervision. **T. Crump:** Conceptualization; investigation; funding acquisition; writing – original draft; writing – review and editing; methodology; resources. **C. J. Brown:** Investigation; writing – review and editing. **M. J. Raval:** Investigation; writing – review and editing. **A. Ghuman:** Investigation; writing – review and editing. **J. Mok:** Conceptualization; investigation; writing – original draft; writing – review and editing.

## FUNDING INFORMATION

This study was supported in kind by Vancouver Coastal Health (VCH) Authority and Providence Healthcare (Vancouver). VCH and Providence Healthcare had no role in developing the methods, data analyses, interpreting the results or manuscript preparation.

## CONFLICT OF INTEREST STATEMENT

The authors report no conflicts of interest.

## ETHICS APPROVAL STATEMENT

The University of British Columbia's Behavioural Research Ethics Board approved the study (H21‐02641).

## PATIENT CONSENT STATEMENT

Each patient has provided written consent.

## Data Availability

Anonymized data is available from the corresponding author.
